# Supplementary material for: Readiness of primary care centres for a community-based intervention to prevent and control noncommunicable diseases in the Caribbean: A participatory, mixed-methods study
Source: PLoS One. 2024 Apr 29;19(4):e0301503. doi: 10.1371/journal.pone.0301503 (PMC11057736; doi:10.1371/journal.pone.0301503)
Supplement: S1 Table — (PDF) [file pone.0301503.s002.pdf]

Supplementary Table 1: Composition of stakeholders by country

	Guyana	Jamaica	Dominica
Ministry of Health	8	7	5
Religious representatives	5	4	6
Academia	4	1	3
Non-government Organisation representatives (e.g. Pan American Organisation)	1	2	2
Total	18	14	16
