# Supplementary material for: Readiness of primary care centres for a community-based intervention to prevent and control noncommunicable diseases in the Caribbean: A participatory, mixed-methods study
Source: PLoS One. 2024 Apr 29;19(4):e0301503. doi: 10.1371/journal.pone.0301503 (PMC11057736; doi:10.1371/journal.pone.0301503)
Supplement: S2 Table — (PDF) [file pone.0301503.s003.pdf]

**Supplementary Table 2: Themes; bridging values; average importance and feasibility ratings and statements for stakeholders based on their response to the prompt *'Factors that will affect the ability of health centres to promote this intervention are…"***

*1a) Guyana*

| **Themes (B = average bridging score)** | **Average Importance Rating*** | **Average Feasibility Rating*** | **Statements rated high on importance and feasibility**** |
|---|---|---|---|
| **Service capacity** (B = 0.17) | 4.30 | 3.45 | 2. Opening times of health centre; **3. Commitment of staff to intervention; 4. Roles and responsibilities of staff; 5. Leadership in health centres;** 7. Small size of health centre buildings; 14. Under-staffing at health centres; 16. Location of the health centre in relation to places of worship; 19. Lack of trained health centre staff to supervise health advocates; 20. Health centre unable to manage referred patients; 21. Lack of trained health centre staff to support intervention; 29. Poor awareness of mental health issues at health centres |
| **Training** (B = 0.33) | 4.58 | 4.08 | **1. Communication between staff and health advocates; 13. Length of training for health advocates; 17. Training of health centre practitioners; 18. Process of incorporation of health advocates in health centers.** |
| **Resources** (B = 0.35) | 4.33 | 3.0 | 6. Financial resources; 22. Lack of access to medicines; 23. Lack of equipment<br>24. Lack of government support for the service; 25. Political Interference; **31. Buy-in of senior Ministry of Health personnel** |
| **Supervision** (B = 0.42) | 4.10 | 4.50 | 8. Small populations served by health centre; 9. Level of non-communicable diseases in local areas; 30. Social workers to address social issues |
| **Socio-cultural factors** (B = 0.71) | 3.84 | 3.73 | 10. Socio-economic circumstances of localities; 11. Gender of health centre practitioners; 12. Ethnicity of health centre practitioners; 15. Trust in modern medicine in rural areas; 27. Education level of the congregations; 28. Cultural beliefs/practices of health advocates<br>**32. Religious leaders' recognition of value of intervention; 33. A. Availability of congregants with skills to be health advocates** |

*1b) Jamaica*

| **Themes (B = average bridging score)** | **Average Importance Rating*** | **Average Feasibility Rating*** | **Statements rated high on importance and feasibility**** |
|---|---|---|---|
| **Resources** (B = 0.14) | 4.27 | 3.77 | **1. Financial resources; 5. Human resources; 6. Clinical and technological resources;** 10. Availability of transportation for health advocates and congregations to health centres; 11. Availability of mobile health services for congregation members |
| **Stakeholder motivation** (B = 0.42) | 4.25 | 3.83 | 8.Level of "buy in" and commitment among congregations; **9. Level of personal responsibility for health among congregations;** 13.Promotion of health advocate role as career boosting opportunity; **14. Community acceptance of intervention** |
| **Community collaboration** (B = 0.68) | 4.23 | 3.70 | N/A |
| **Service capacity** (B = 0.73) | 4.54 | 3.79 | N/A |
| **Health's advocate role** (B = 0.74) | 4.57 | 4.00 | **2. Clarity of roles and responsibilities; 3. Consistency of feedback regarding success; 4. Focus on prevention as well as supporting persons living with non-communicable diseases;** 7.Accountability of project staff; **17. Consistency of monitoring and evaluation of intervention processes and performance of health advocates** |

1c) Dominica

| Themes (B = average bridging score) | Average Importance Rating* | Average Feasibility Rating* | Statements rated high on importance and feasibility** |
|---|---|---|---|
| **Service capacity** (B = 0.17) | 4.05 | 3.59 | 1. Health centres are usually busy; 3. Limited clinic hours for patients; 6. Sickness of staff; **9. Confidentiality of records;** 14. Overworked personnel at the health centre; 18. Staff will work overtime for additional tasks; 19. Understaffing at health centres; 20. Staff are very busy managing current caseload; 29. Overburdened staff; 30. Difficulty handling emergencies; 31. Poor time management; 32. Staff shortages are constant; 33. Nurses are on call for emergencies which can mean no time for other services; **34. Excited and eager staff;** 36. Number of staff at the health centre;**41. Attitudes of health workers towards non health workers** **44. Communication between the health workers and the community;** 46. Limited opening hours; 50. Not problematic for health centres to promote this intervention; **52. Commitment of staff** |
| **Training** (B = 0.42) | 4.21 | 3.92 | 7. Inadequate stationary; **15. Help with training; 28. Inadequate human resources; 38. Experience and expertise of health professionals** **40. Training of health advocates;** 43. Incentives for health care workers;47. Inefficient use of resources. |
| **Resources** (B = 0.29) | 4.18 | 3.90 | 16. Limited financial resources; **17. Special budget for the intervention;** 25. Churches may not have equipment to use in this program; 26. Economics play a crucial role; 27. Lack of teaching materials; **37. Availability of equipment** **45. Materials for health advocates** **51. Lack of medical equipment** |
| **Health Centre – Place of Worship collaboration** (B = 0.62) | 4.54 | 3.79 | 4. Electricity failures; 11. Understanding history and culture of Kalinago; 12. Pollution in the local environment; **13. Eating too much junk food; 22. Integrate health and churches programmes; 23. Church and health** |

| | | | |
|---|---|---|---|
| | | | **teams to generate activities jointly; 24. Health workers in congregations as contact persons; 42. Acceptability of the programme by the Ministry of Health;** 49. Too much talk and no action |
| **Church receptiveness** (B = 0.73) | 3.58 | 3.00 | 2. The health centre is far from patients' homes; 5. Inclement weather; 8. Lack of transportation; 10. Rules of religion; 21. Churches may not see this be within their religious programme; 35. Location of health centre in the territory; **39. Willingness of congregants to participate;** 48. Lack of interest in health programs |
