# Supplementary material for: Readiness of primary care centres for a community-based intervention to prevent and control noncommunicable diseases in the Caribbean: A participatory, mixed-methods study
Source: PLoS One. 2024 Apr 29;19(4):e0301503. doi: 10.1371/journal.pone.0301503 (PMC11057736; doi:10.1371/journal.pone.0301503)
Supplement: S3 Table — (PDF) [file pone.0301503.s004.pdf]

**Supplementary Table 3. Readiness assessments of health centres: summary of enablers and barriers to promote the embedding of places of worship into the primary care pathway**

| | GUYANA | | | | | | JAMAICA | | | | DOMINICA (KALINAGO TERRITORY) | |
|---|---|---|---|---|---|---|---|---|---|---|---|---|
| | **Canal No. 1** | **Tuschen** | **Versailles** | **Meten-Meer-Zorg** | **Den Amstel** | **Parika** | **Riversdale- Rural** | **Yallahs- Rural** | **Olympic Gardens- Urban** | **Norman Gardens- Urban** | **Salybia** | **Mahaut River** |
| | 4000 | 15000 | 3000 | 3000 | 6000 | 15000 | 15000 | 39000 | 25000 | 15000 | 2000 | 2000 |
| **General infrastructure, amenities, accessibility** | Relatively small and functional, with 2 examination rooms; no dedicated dental or pharmacy rooms. Open weekdays only. Water, electricity and sanitation facilities for patients and staff available. No working telephone or internet; staff use their personal phones. Residents use public or personal transportation to the health centre; transportation to next level of care is requested from regional hospital and dependent on availability of those services. Health centre located on the main road. | Relatively large and functional, with 4 examination rooms and capacity for minor surgeries and dental services. Open weekdays only. Electricity, portable water and sanitation facilities available; staff provide potable water for themselves and patients. No telephone, internet or transportation services; patients take a taxi to next level of care, accompanied by a nurse if available. Health centre difficult to access, with unpaved roads. | Functional, with 3 examinations rooms; no dedicated dental or pharmacy rooms. Open weekdays only. Electricity, water and sanitation facilities available. Telephone is available, but not internet. Patients use public or private transportation to the health centre; transportation for referral is requested from regional hospital ambulance service. Health centre located on the main road. | Small and functional. One examination room. Outdoor waiting area, with little to no space to accommodate increased patient inflow from CONTACT. Located close to the main road so fairly accessible to residents via public transportation. Facilities are open on weekdays but doctors not always available. Electricity is available but no backup power. Toilets are currently broken with no running water. Landline phone but no internet available. | Small and functional. Two examination rooms. Limited waiting area to accommodate additional patients from CONTACT. Located on the main road so is accessible via public transportation. Electricity available but no backup power. Potable and drinking water always available as located next door to water utility. Landline telephone available but no internet. | Large and functional. Four examination rooms available. Serves a relatively large population, so limited space available to accommodate patients from CONTACT. Electricity available but no backup generator. Potable water and backup available. Staff have to buy their own drinking water. No telephone available. | Small but functional for community served approximately 15000 persons, 3 rooms allocated for specific clinical services, small waiting area, limited access to public transportation. No internet service, clinic has a landline. Electricity and a small back up generator which was donated to the facility. | Small infrastucture , inadequate for community needs, community served approx 39000 persons. Dental clinic and pharmacy on site, lab personnel visit twice per month , four treatment rooms for physicians, small crowded waiting area . On the main road in the community with easy access by public transportatio. Itranet access in the pharmacy not the general health centre. Landline operational. Electricity and potable water. Facility has private rooms examinatiuon that allows for visual and audio privacy. | Infrastructure for clinical services including a small pharmacy and phlebotomy room , 6 examination rooms,inadequate for community served of 25000 persons, moderate sized waiting area that is crowded, In an area that has easy access to public transportation which is within walking district. Landline operational, pharmacy has intranet to corporate offices. Transportation available to urgent care needs. Electricity and potable water avaialble. | Adequate infrastucture for community needs , community population approx 15000, relatively small waiting area but adequate There are 6 examination rooms . Public transportation is within walking distance . No internet service but landline operational but sited in the doctors office. electricity and potable water avaiable. | Large and functional. 7 treatment rooms which includes 2 dental rooms and 2 pharmacy rooms. There is space to accommodate increase inpatient flow from CONTACT and for secure storage. Poor access to public transport. Health facility is opened daily but nurse on call 24 hrs for emergencies. Water and electricity are available. There is no internet or landline phone. Health staff use personal mobile phone to communicate with clients,other health care providers and community. | Small and functional.3 treatment rooms and a small pharmacy area. Limited space to accommodate increase inpatient flow from CONTACT or for secure storage. Poor public transport. Health facility is opened daily with limited opening hours and nurse on call 24 hrs for emergencies. Water and electricty are available. Internet and landline phone available. |

| | | | | | | | | | | | |
|---|---|---|---|---|---|---|---|---|---|---|---|
| **Strong leadership at HC/district levels** | Staff are employed by the Ministry of Health. Placement of staff is decided centrally at the Ministry level. Once a month staff meet with regional health authorities to discuss needs. All drugs, equipment and other supplies are officially provided by the Ministry of Health. However, many donations are also received from members of the public. | Staff are employed by the Ministry of Health. Placement of staff is decided centrally at the Ministry level. Once a month staff meet with regional health authorities to discuss needs. All drugs, equipment and other supplies are officially provided by the Ministry of Health. However, many donations are also received from members of the public. | Staff are employed by the Ministry of Health. Placement of staff is decided centrally at the Ministry level. Once a month staff meet with regional health authorities to discuss needs. All drugs, equipment and other supplies are officially provided by the Ministry of Health. However, many donations are also received from members of the public. | Staff are employed by the Ministry of Health. Placement of staff is decided centrally at the Ministry level. Once a month staff meet with regional health authorities to discuss needs. All drugs, equipment and other supplies are officially provided by the Ministry of Health. However, many donations are also received from members of the public. | Staff are employed by the Ministry of Health. Placement of staff is decided centrally at the Ministry level. Once a month staff meet with regional health authorities to discuss needs. All drugs, equipment and other supplies are officially provided by the Ministry of Health. However, many donations are also received from members of the public. | Staff are employed by the Ministry of Health. Placement of staff is decided centrally at the Ministry level. Once a month staff meet with regional health authorities to discuss needs. All drugs, equipment and other supplies are officially provided by the Ministry of Health. However, many donations are also received from members of the public. | Staff employed by the regional health authority, public helath nurse provides overall supervision of the centre. She reports to the senior public helath nurse who is responsible for that district. Staff have clear job descriptions. No autonomy at the health centre level | Staff employed by the regional health authority, Health centre managed by the public health nurse, there is one supervisor (Snr Public Health Nurse)for each health district. The PHN supervises the midwives who in turn supervises the CHWs. Nutritionist and mental health worker assigned to clinic | Decisions are made at the regional health authority to which staff are employed . Public health nurse manages the health centre and prvides task assignments for staff. | Staff employed by regional helath authority, the senior public health nurse at this centre is responsible for several other centres. | Ministry of Health, employs staff. Decisions about community programs made by management team who are employed at the health district level, not at the Kalinago Health Centre level. Involves District Medical Officer who is head of the health district, Community Health Nurse who supervises all nurses in the district, Family Nurse Practioner, Environmental Officer and Pharmacist. Centre built and owned by Kalinago Council and leased by Ministry of Health. Ministry of Health provides furnishings and supplies, however, the Kalinago Council also provides infrastructural support when they | Ministry of Health, employs staff. Decisions about community programs made by management team who are employed at the health district level, not at the Kalinago Health Centre level. Involves District Medical Officer who is head of the health district, Community Health Nurse who supervises all nurses in the district, Family Nurse Practioner, Environmental Officer and Pharmacist. Centre built and owned by Kalinago Council and leased by Ministry of Health. Furnishings, supplies, medical equipment provided by the Ministry of Health. |

| | | | | | | | | | | | | |
|---|---|---|---|---|---|---|---|---|---|---|---|---|
| | | | | | | | | | | | receive donations. | |
| **Services offered** | Weekly family planning, antenatal care, child health services, HIV testing and counselling, and prevention of mother-to-child transmission. Chronic disease clinics are held twice per month, and minor surgeries are also done when needed. Preventative services include health promotion, risk factor detection, early detection/screening, support for home based care and palliative care services; however, these services are not provided regularly. | Weekly antenatal care, HIV testing and counselling, HIV/AIDS care and support and chronic disease services including diabetes clinic. Family planning and child health clinics are held twice per month. Minor surgeries including sutures and abscess drainage are performed. Primary prevention and health promotion in the form of counselling and health talks is offered, along with risk factor detection, early screening, risk factor and disease management, palliative care and support for self help and home based care, although they are not done regularly. | Weekly family planning, antenatal care, child health services, basic emergency obstetrics and chronic disease services including diabetes clinic. Outpatient dressings are done daily. Primary prevention and health promotion is offered, along with risk factor detection, early screening and disease management, surveillance and palliative care services. | Daily family planning clinic available; weekly antenal clinic, child health clinic, HIV testing and counselling, HIV/AIDS care and support, chronic disease clinic and diabetes clinic. Prevention of mother-to-child transmission services also offered. No mental health clinic. Staff were not aware of the availability or use of any NCD protocols. Early detection services included anthropometric measurements, digital rectal exam, breast cancer screening by palpation, blood glucose measurement, blood pressure testing and urine protein testing, when equipment available. Health promotion activities are also done. | Family planning clinic is available twice monthly, antenatal clinics once per month, child clinics two per month, HIV clinics once a week, PMTCT services and chronic disease clinics weekly. Early detection and risk factor screening are available. Anthromorphic measurements, digital rectal examination, breast cancer screening by palpation, blood glucose measurement, HbA1c testing and blood pressure testing are the screening tests available. Health pomotion activities are also done. | Family planning clinic available once weekly, antenatal care twice weekly, basic emergency obstetrics, HIV services once a week, diabetes clinic once per week, chronic disease clinic once per week and mental health clinic once a month. Child health services are also available. | weekly clinics diabetes , chronic disease, child health and antenatal services facilitated by two doctors and a registered nurse who visit weekly. Nutrition and mental health clinics held once monthly. | Diabetes and chronic disease clinics twice per week , mental health, antenatal, child health and obgyn/family planning services 3 times per month. Vaccinations and wellness clinic monthly | Curative clinics are held weekly. The mental health clinics are held three times per month. The Health educator visits monthly. Also provides foot screening in the chronic disease clinics this is done by trained CHW, however not consistently. | Chronic disease clinics held twice weekly, family helath clinics once weekly Nutrition services once monthly. Phlebotomist visit twice per month. | Weekly maternal & child heath clinics. Daily chronic disease clinics, quarterly diabetes clinic & monthly hypertensive clinic, specialist run mental health clinic on request. Preventative services include nurse led screening of CVD risk factors (blood pressure screening, fasting blood sugar, healthy lifestyles), cervical smears, support for home based care for the elderly, health promotion sessions in waiting area. | Weekly maternal & child heath clinics. Daily chronic disease clinics, quarterly diabetes clinic & monthly hypertensive clinic, specialist run mental health clinic on request. Preventative services include nurse led screening of CVD risk factors (blood pressure screening, fasting blood sugar, healthy lifestyles), cervical smears, support for home based care for the elderly, health promotion sessions in waiting area. |

| | | | | | | | | | | | | |
|---|---|---|---|---|---|---|---|---|---|---|---|---|
| **Essential medicines** | Variable with a history of drug shortfall for many common drugs including aspirin. Insulin and a few other chronic disease medications not available. | Variable with a history of drug shortfall for drugs such as aspirin and metformin. Many chronic disease drugs unavailble including drugs for depression and asthma. | Good availability of essential medication; however, most have a history of drug shortfall | Diabetic, hypertensive, cardiovascular, depression, infectious disease, pain/inflammatory, central nervous system and stomach ulcer medication are all supplied to the health centre but there are frequent shortfalls throughout the year for most of them. | Diabetic, hypertensive, cardiovascular, depression, infectious disease, pain/inflammatory, central nervous system and stomach ulcer medication are all supplied to the health centre but there are frequent shortfalls throughout the year for most of them. | Diabetic, hypertensive, cardiovascular, depression, infectious disease, pain/inflammatory, central nervous system and stomach ulcer medication are all supplied to the health centre but there are frequent shortfalls throughout the year for most of them. | No inhouse pharmacy access through community pharmacy | Small pharmacy onsite that provides most routinely prescribed drugs | Small pharmacy onsite that provides most routinely prescribed drugs | access to drugs through community pharmacy. In proximity of the HC | Variable with small quantities for some conditions e.g. depression meds; or not supplied by government e.g. statins. | Variable with small quantities for some conditions e.g. depression meds; or not supplied by government e.g. statins |
| **Technical tools to aid front line delivery** | Variable access to basic equipment, with nurse having to use personal glucometer on patients. Patients are referred to regional hospital for laboratory facilities. | Variable access to basic equipment, with no available thermometer. Patients are referred to regional hospital for laboratory services. | Access to basic equipment, including thermometer, stethoscope, blood pressure apparatus and scales. Patients are referred to regional hospital for laboratory services. | Sphygmomanometers, glucometers, scales, stadiometers available. However, glucometer strips not always available. No lab technologists available to take off blood samples. | Sphygmomanometers, glucometers, scales, stadiometers available. However, glucometer strips not always available. No lab technologists available to take off blood samples. | Sphygmomanometers, glucometers, scales, sbut no thermometer available. However, glucometer strips not always available. No lab technologists available to take off blood samples. | Basic - scales, urinalysis strips and pregnacy tests. Blood pressures and glucose test are done by visiting nurse | Basic - sphygs, glucometers , scales , POC HbA1c, and preganacy tests; samples collected twice per month by visiting phlebotomist and assayed at the national public health lab; staff uses their personal stethoscope | Basic - sphygs, glucometers , scales, thermometors. Phlebotomist visits weekly for blood draws ( mainly from the antenatal clinic which are processed at the National public helath lab. | Basic - scales Physician and nurses use their own stethoscopes. . Blood pressure machine available but there is limited cuff size. Thermometers available in Maternal and child health section | Access to basic equipment (OMRON, gluconometers, scales etc). Blood samples are drawn twice a month and then sent to the public hospital or private laboratory for analysis. | Access to basic equipment (OMRON, gluconometers, scales etc). Blood samples are drawn twice a month and then sent to the public hospital or private laboratory for analysis. |
| **Health information systems** | Paper-based. Patient charts are organised and updated monthly when the patient attends clinic. | Paper-based. Patient charts not available; patient cards are used instead, which are not organised or updated regularly. | Paper-based. Patient charts are organised and updated regularly. | Paper-based. Patient logs are adequately maintained and are used for requisition of supplies. | Paper-based. Patient logs are adequately maintained and are used for requisition of supplies. | Paper-based. Patient logs are adequately maintained and are used for requisition of supplies. | Paper based records. Dockets that are filed and accessed by visiting staff. Monthlysurveillance reports done by PHN and submitted to the St Catherine health department | paper based records - dockets filed by medical records clerk. Monthly reports done by PHN and submitted to St Thomas Health Dept | Paper based logs/dockets filed by medical records clerk , collated by public helath nurse and reports sent to MOH surveillance unit A central database is to be installed. | Paper based dockets managed by the records clerk | Paper-based; NCD register well maintained and used for monthly reports. | Paper-based; NCD register well maintained and used for monthly reports. |

| | | | | | | | | | | | | |
|---|---|---|---|---|---|---|---|---|---|---|---|---|
| **Implementation of NCD protocols** | Aware of NCD protocols, which are only implemented partially, but are not being monitored. | Only aware of diabetes protocol, which is implemented partially, but not being monitored. Surveillance being done for NCDs and reported on special Ministry of Health forms. | aware of protocols for diabetes and chronic respiratory disease; only implemented partially, but are not being monitored. | Staff are not aware of the existence of NCD protocol and thus none are officially implemented and monitored. | Staff are not aware of the existence of NCD protocol and thus none are officially implemented and monitored. | Could not comment on availability of NCD protocols (not aware) | Not aware of NCD protocols | NCD protocols on Diabetes , CVD and respiratory diseases avaialble and fully implemented. Protocls on tobacco dependence partially implemented. Not aware of cancer protocols | NCD guidelines available, not sure of cancer protocols, however diabetes and chronic resp disease protocols fully implemented , CVD and tobacco partially. | There are NCD guidelines available ?No mention of implementati on of the guidelines | National hypertension and diabetes manual. Evaluation done by quarterly reports submitted to the Family Nurse Practioner e.g frequency of diabetic foot clinics. | National hypertension and diabetes manual. Evaluation done by quarterly reports submitted to the Family Nurse Practioner e.g frequency of diabetic foot clinics. |
| **Referral pathways to specialist care** | Doctors at the health centre would refer patients to the Regional or to the Central Referral Hospital, depending on whether services are known to be available at the regional hospital. | Doctors at the health centre would refer patients to the Regional or to the Central Referral Hospital, depending on whether services are known to be available at the regional hospital. | Doctors at the health centre would refer patients to the Regional or to the Central Referral Hospital, depending on whether services are known to be available at the regional hospital. | Doctors at the health centre would refer patients to the Regional or to the Central Referral Hospital, depending on whether services are known to be available at the regional hospital. | Doctors at the health centre would refer patients to the Regional or to the Central Referral Hospital, depending on whether services are known to be available at the regional hospital. | Doctors at the health centre would refer patients to the Regional or to the Central Referral Hospital, depending on whether services are known to be available at the regional hospital. | Medical officers provide referral to specialist or tertiary care services as indicated | Patients referred by medical officers to tertiary facilities for treament mainly the kingston public hospital and if necessary the University hospital | Patients referred by medical officers to tertiary facilities for treament mainly the kingston public hospital and if necessary the University hospital | Patients referred by medical officers to tertiary facilities for treament mainly the kingston public hospital | District Medical Officer refers to specialists at the main hospital on Island or to private specialist | District Medical Officer refers to specialists at the main hospital on Island or to private specialist |
| **Referral pathways to social care** | No social worker available, thus persons are referred by doctors on needs basis to the regional hospital's social worker. | No social worker available, thus persons are referred by doctors on needs basis to the regional hospital's social worker. | No social worker available, thus persons are referred by doctors on needs basis to the regional hospital's social worker. | No social worker available, thus persons are referred by doctors on needs basis to the regional hospital's social worker. | No social worker available, thus persons are referred by doctors on needs basis to the regional hospital's social worker. | No social worker available, thus persons are referred by doctors on needs basis to the regional hospital's social worker. | Mental health officers visit once monthly , CHAs provide some support in the community | Mental health officer assists in social services at the clininc level, community health aides make referrals at the community level | Mental health officer and health educators provide some social support . | None | Referral done by Community Health Nurse for welfare/social services, and social support programmes such as the "Yes We Care" for inidviduals who are not able to take care of themselves due to disability and other morbid | Referral done by Community Health Nurse for welfare/social services, and social support programmes such as the "Yes We Care" for inidviduals who are not able to take care of themselves due to disability and other morbid |

| | | | | | | | | | | | | |
|---|---|---|---|---|---|---|---|---|---|---|---|---|
| | | | | | | | | | | | conditions (stroke, injuries etc.). | conditions (stroke, injuries etc.). |
| **Regular monitoring and evaluation reports** | Health centre staff report to the Senior Health Visitor (stationed at regional hospital). Meetings are held monthly with senior staff (medex/nurse in charge) reporting on patients seen and services provided. CHW report to senior staff. | Health centre staff report to the Senior Health Visitor (stationed at regional hospital). Meetings are held monthly with senior staff (medex/nurse in charge) reporting on patients seen and services provided. CHW report to senior staff. | Health centre staff report to the Senior Health Visitor (stationed at regional hospital). Meetings are held monthly with senior staff (medex/nurse in charge) reporting on patients seen and services provided. CHW report to senior staff. | Health centre staff report to the Senior Health Visitor (stationed at regional hospital). Meetings are held monthly with senior staff (medex/nurse in charge) reporting on patients seen and services provided. CHW report to senior staff. | Health centre staff report to the Senior Health Visitor (stationed at regional hospital). Meetings are held monthly with senior staff (medex/nurse in charge) reporting on patients seen and services provided. | Health centre staff report to the Senior Health Visitor (stationed at regional hospital). Meetings are held monthly with senior staff (medex/nurse in charge) reporting on patients seen and services provided. CHW report to senior staff. | CHW report to PHN who updates surveillance data for parish health department. The surveillance data is sent to the parish health department monthly. | PHN sends surveillance reports to parish health department monthly. | All Staff except medical officers report to PHN who updates surveillance data which is sent to the Ministry of Health monthly. | Senior public health nurse who manages the centre and several others in Zone 1 collates data for the health centres.She is responsible for monthly reports to the MOH | Nurses provide reports to their supervisor - Community Health Nurse, and newly diagnosed cases are discussed at monthly health centre meetings. Chronic disease management, caseload management discussed at quarterly meetings. | Nurses provide reports to their supervisor - Community Health Nurse, and newly diagnosed cases are discussed at monthly health centre meetings. Chronic disease management, caseload management discussed at quarterly meetings. |
| **HEALTH WORKERS** | | | | | | | | | | | | |

| | | | | | | | | | | | | |
|---|---|---|---|---|---|---|---|---|---|---|---|---|
| **Sufficient staff to manage patient flows (dr/patient ratios? Nurses, CHWs etc?)** | Inadequate - 1 doctor, 1 nurse aide, 1 midwife, 1 community health worker, 1 visiting dental surgeon twice/month; to serve a population of approximately 3000. | Inadequate - 1 doctor, 2 nurse aides, 1 staff nurse, 1 midwife, 1 dental surgeon, 1 dental nurse, 1 community health worker, 1 prevention of mother-to-child transmission staff; to serve a population of over 6000. | Inadequate - 1 doctor, 1 medex, 1 midwife, 1 community health worker, 1 nursing assistant, 1 clinic attendant. POP SIZE NEEDED | Staff to patient ratios are inadequate. Staff included 1 doctor, 1 medex, 1 midwife, 1 nurse aid and 1 community health worker, serving a population of 3000. | Staff to patient ratios are inadequate. Staff include 1 doctor, 1 registered nurse, 1 nurse aide, 1 midwife. Patients reported that a second doctor being available would increase efficiency of the health centre. POPULATON SERVED? | Staff to patient ratios are inadequate. Staff include 1 doctor, 1 nurse, 2 midwives, 1 community health worker serving a population of greater than 5000. | Facilty has one public health nurse, one midwife and 4 CHW and 1 medical records clerk, servinf a popualtion of 15000 | Facility has 2 doctors, 2 PHN, 1 RN, 2 midwives , 4 CHW , 1 mental health worker and a nutritionist assigned .Serve a population of 39000. Poor staff: patient ratio. Centre sees on average 70 patients per day in the chronic disease clinic | Facilty has 3 doctors, 1 PHN, 1RN, 1 enrolled nurse , 5 community health aides, 2 medical records clerk. Serve a population of 25000. Poor ratio of HCP: patient. The chronic disease clinic has approx 2500 persons registered with 80% uptake. patients wait approx 1.5hrs to be seen | One medical officer full time, two others visit when necessary, 1 staff nurse, 1 midwife , 3 community health aides. Serve a popuation of 1500 | Inadequate - visiting District Medical Officer twice monthly; visiting family nurse practitioner twice monthly; 1 nurse, 1 Community Health Aide. Nurses provide emergency care which constrains regular services. Serve approximately popultaion of 800-1000 | Inadequate - visiting District Medical Officer twice monthly; visiting family nurse practitioner twice monthly;1 nurse, 1 Community Health Aide. Nurses provide emergency care which constrains regular services. Seve approximattely popultaion of 800-1000 |

| | | | | | | | | | | | | |
|---|---|---|---|---|---|---|---|---|---|---|---|---|
| **Training of health centre staff in NCDs** | All staff initially trained, with most trained in NCD management and health promotion. There are no regularly scheduled training sessions. | All staff initially trained, with most trained in NCD management and health promotion. There are no regularly scheduled training sessions. | All staff initially trained, with most trained in NCD management and health promotion. There are no regularly scheduled training sessions. | All staff initially trained, with most trained in NCD management and health promotion. There are no regularly scheduled training sessions. | All staff initially trained, with most trained in NCD management and health promotion. There are no regularly scheduled training sessions. Senior staff provide training to junior staff when needed. | All staff initially trained, with most trained in NCD management and health promotion. There are no regularly scheduled training sessions. Doctor in Charge provides ad hoc training to junior staff. | Monthly inservice training for nurses done by the region. Community health workers not trained in NCDs; their focus is mainly on maternal and child health | Monthly inservice training for nurses done by the region | Monthly inservice training for nurses done by the region | Monthly inservice training for nurses done by the region | Monthly in-service training, done by Community Health Nurse, District Medical Officer, Family Nurse Practioner, Nutritionist or other trained professionals. Workshops and symposiums by Pan American Health Organisation, Ministry of Health and other health organisations. | Monthly in-service training, done by Community Health Nurse, District Medical Officer, Family Nurse Practioner, Nutritionist or other trained professionals. Workshops and symposiums by Pan American Health Organisation, Ministry of Health and other health organisations. |
| **Training of health centre staff in communication** | Inadequate. No regularly scheduled training in communication | Inadequate. No regularly scheduled training in communication | Inadequate. No regularly scheduled training in communication | Inadequate. No regularly scheduled training in communication | Inadequate. No regularly scheduled training in communication | Inadequate. No regularly scheduled training in communication. | No regularly scheduled training in communication. | No routine staff training , but some training conducted during the regional nurses meeting | Ad hoc training in this area | No training , medical officers provides some training for other staff as necessary | Inadequate, last training in communication was 2 years ago. | Inadequate, last training in communication was 2 years ago. |

| | | | | | | | | | | | | |
|---|---|---|---|---|---|---|---|---|---|---|---|---|
| Willingness and motivation to support CONTACT | Yes - appreciated the potential benefits of CONTACT. | Yes - appreciated the potential benefits of CONTACT. | Yes - appreciated the potential benefits of CONTACT. | Yes but felt that lack of resources might be a barrier | Yes but felt that lack of resources might be a barrier | Yes but felt that lack of resources might be a barrier. | Yes as patients often do not attend due to lack of transport. Community health workers, however, are not trained to do BP. On clinic days however may not be feasible for supervision of HA or for HAs to assist in the clinic as the nurse may not want someone to come in. If assistance is needed from the HA it would be for health education.The PHN is not from the community so would not be able to supervise in the community setting. | The intervention would be limited by the fact that there is an overcrowded facility at most times with a small number of staff to cover the patient load. The presence of the health advocate would increase the workload of the already burdened staff if tehy have to supervise the HA. | Support for health advocates through talks at the churches. Health advocates can aid with health promotion sessins in the clinic as helath educators comes monthly | Not feasible for staff to supervise health advocates due to excessive workload. Staff not familiar with the community and cannot work on weekends. However health centre-church collaboration would promote holistic health | Willingness to provide support with health promotion education and blood pressure screening | Willingness to provide support with health promotion education and blood pressure screening |
| Clarity of roles and responsibilities of all practitioners | Organisational chart for facility is not available; staff did not receive training/orientation on their roles | Organisational chart present and staff aware of their core duties and functions. | Organisational chart present and staff aware of their core duties and functionc. | Staff are aware of their roles and responsibilities. An organisation chart available. | Staff are aware of their roles and responsibilities but no organisational chart available | Staff are aware of roles and responsibilities but no official delegation of duties or job descriptions or organisation chart available. | Staff aware of roles and responsibilities | Staff aware of roles and responsibilities | Staff aware of roles and responsibilities | Staff aware of roles and responsibilities | Staff aware of roles and responsibilites. | Staff aware of roles and responsibilites . |

| | | | | | | | | | | | | |
|---|---|---|---|---|---|---|---|---|---|---|---|---|
| **Health Centre managers have the autonomy to support CONTACT** | Approval is needed from Ministry of Health and Regional Health Officer, without it, health centre does not have the autonomy to support CONTACT. | Approval is needed from Ministry of Health and Regional Health Officer, without it, health centre does not have the autonomy to support CONTACT. | Approval is needed from Ministry of Health and Regional Health Officer, without it, health centre does not have the autonomy to support CONTACT. | Approval is needed from Ministry of Health and Regional Health Officer, without it, health centre does not have the autonomy to support CONTACT. | Approval is needed from Ministry of Health and Regional Health Officer, without it, health centre does not have the autonomy to support CONTACT. | Approval is needed from Ministry of Health and Regional Health Officer, without it, health centre does not have the autonomy to support CONTACT. | Approvals needed from the regional health authority | Approvals needed from the regional health authority | Approvals needed from the regional health authority | Approvals needed from the regional health authority | Approvals required by Ministry of Health, Kalinago Council | Approvals required by Ministry of Health, Kalinago Council |
| **COMMUNITY COLLABORATIONS** | | | | | | | | | | | | |
| **Community engagement/health promotion** | Very limited engagement with community; no history of collaborations with places of worship. Health promotion is only done at the health centre. | Limited community engagement with some health promotion activities held at places of worship; most health promotion is conducted at the health centre. | Very limited engagement with the community; no history of collaborations with places of worship. Health promotion mostly done at the health centre. | The health centre engages with places of worship in their catchment area. | No past collborations with places of worship and not aware of any current collaborations or community acitvities for health promotion | No past collborations with places of worship and not aware of any current collaborations or community acitvities for health promotion | Yes via community health workers but limited with the focus on WHAT? | Community health worker work on maternal and child health with communities. Health educators visit monthly and provide education at the Diabetes or chronic disease clinic. Ministry of Health and PAHO health promotion flyers are prominent in the clinics. Limited engagement in community health fairs. None with schools | Health educators assist in community health fairs, in collaboration with local NGOs such as the Heart Foundation and Diabetes association. | Limited community engagement. Aware of only national programmes and that churches provide health fairs and a food programme | Numerous with schools, churches, community events, jointly organised. Health promotion sessions at the health centre staff every Thursday. | Numerous with schools, churches, community events, jointly organised. Health promotion sessions at the health centre staff every Thursday. |

| | | | | | | | | | | | |
|---|---|---|---|---|---|---|---|---|---|---|---|
| **Provision of ongoing care of patients with chronic disease in the community** | Home based care by community health worker is offered sometimes. | Home based care is offered by community health worker when needed, although not required by many patients. | Home based care by community health worker. | Community health workers go out into the field to see patients at home as needed. | At the time of the PHC evaluation, health centre reported not having CHW. A new CHW later joined (and was involved in CONTACT) and reported doing home visits as needed, but provides limited, basic care, mostly vitals and dressings. | Community health workers go out into the field to see patients at home as needed. | Community health workers provide limited service, most not trained to do BP checks | Community health workers provide some follow up in the community | Community health workers provide some follow up and referral to healthcentres . They were trained to provide community intervention and referral , they currently operate mainly at the family heatlh level. Very few are trained in NCD promotion. | Community healthworkers provide maternal and child health services in the community | Home based care by nurse, community health aide | Home based care by nurse, community health aide |
| **GOVERNMENT SUPPORT** | | | | | | | | | | | |

| | | | | | | | | | | | | |
|---|---|---|---|---|---|---|---|---|---|---|---|---|
| **Support for integrating community programmes into primary care pathway, including financing mechanisms** | No regular support available for these kinds of programmes. Consideration may be given if special requests are made. | No regular support available for these kinds of programmes. Consideration may be given if special requests are made | No regular support available for these kinds of programmes. Consideration may be given if special requests are made | No regular support available for these kinds of programmes. Consideration may be given if special requests are made | No regular support available for these kinds of programmes. Consideration may be given if special requests are made | No regular support available for these kinds of programmes. Consideration may be given if special requests are made | Limited support for community interventions, financing has to be sought outside the public sector. National Health fund provides health promotion and health fairs at intervals in many communities, in collaboration with NGOs | Health educators assist in community health fairs. Public health nurse assists in annual medicals. No financing. The National Health Fund (an agency of the MOH) provides health promotion and health fairs at intervals in many communities , most in collaboartion with NGOs | National Health Fund provides health promotion and health fairs at intervals in many communities, in collaboration with NGOs. The health educator will participate in community health fairs. No finance available from the region | None that they are aware of | Inadequate support for community programmes run by health centre to target lifestyles. No specific funding stream | Inadequate support for community programmes run by health centre to target lifestyles. No specific funding stream |

| | | | | | | | | | | | | |
|---|---|---|---|---|---|---|---|---|---|---|---|---|
| **Support for NCD training, continuing training etc** | MOH budget for 'Training' but at the level of the PHC, any training is usually one-off. | MOH budget for 'Training' but at the level of the PHC, any training is usually one-off. | MOH budget for 'Training' but at the level of the PHC, any training is usually one-off. | MOH budget for 'Training' but at the level of the PHC, any training is usually one-off. | MOH budget for 'Training' but at the level of the PHC, any training is usually one-off. | MOH budget for 'Training' but at the level of the PHC, any training is usually one-off. | Yes at the regional level | Yes at the regional level | Yes at the regional level | Regional Inservice training for nurses | Monthly in-service training, done by Community Health Nurse, District Medical Officer, Family Nurse Practioner, Nutritionist or other trained professionals. Workshops and symposiums by Pan American Health Organisation, Ministry of Health and other health organisations. | Monthly in-service training, done by Community Health Nurse, District Medical Officer, Family Nurse Practioner, Nutritionist or other trained professionals. Workshops and symposiums by Pan American Health Organisation, Ministry of Health and other health organisations. |
| **Support for use of locally relevant tools, information systems and guidelines** | Posters and some guidelines mounted on walls, provided by MoH and made by health centre staff. No diagnostic tools such as risk scores or support for developing information systems | No posters/guide lines available on walls, unable to tell if any present at PHC. No diagnostic tools such as risk scores or support for developing information system | Posters seen on walls, provided by MOH, unsure what protocols exist and if they are being utilised. No diagnostic tools such as risk scores or support for developing information system | No protocols in place but staff deliver tailored services based on the needs of the population. They also create posters with information for patients about common diseases that are seen at the health centre. No diagnostic tools such as risk scores or support for developing information system | No protocols in place but staff deliver tailored services based on the needs of the population. They also create posters with information for patients about common diseases that are seen at the health centre, along with those provided by MOH. No diagnostic tools such as risk scores or support for developing information system | No protocols in place but staff deliver tailored services based on population needs and available resources - posters from MOH.No diagnostic tools such as risk scores or support for developing information systems | Posters on chronic diseases,mounted , posters are from pharmaceutical companies , MOH and PAHO. No DIAGNOSTIC TOOLS such as risk scores. | Knowledge of guidelines manuals which are stored for and reference ease of access, posters mounted which are supplied by varying entities such as the NHF, PAHO and pharmaceutical companies . No DIAGNOSTIC TOOLS such as risk scores | Protocols are avaiable and utilized in most instances . No DIAGNOSTIC TOOLS such as risk scores | Protocols exist, not sure where. No reports that they are utilized . No DIAGNOSTIC TOOLS such as risk scores | Protocols available for hypertension, diabetes, maternal and child health which are implemented and discussed with Community Health Nurse at monthly health centre meetings. | Protocols available for hypertension, diabetes, maternal and child health which are implemented and discussed with Community Health Nurse at monthly health centre meetings. |

| | | | | | | | | | | | | |
|---|---|---|---|---|---|---|---|---|---|---|---|---|
| | Regional staff are able to make decisions that have no budgetary implications without input from central government. Larger decisions that would affect the budget would need to pass through the Ministry of Health | Regional staff are able to make decisions that have no budgetary implications without input from central government. Larger decisions that would affect the budget would need to pass through the Ministry of Health | Regional staff are able to make decisions that have no budgetary implications without input from central government. Larger decisions that would affect the budget would need to pass through the Ministry of Health | Regional staff are able to make decisions that have no budgetary implications without input from central government. Larger decisions that would affect the budget would need to pass through the Ministry of Health | Regional staff are able to make decisions that have no budgetary implications without input from central government. Larger decisions that would affect the budget would need to pass through the Ministry of Health. | Regional staff are able to make decisions that have no budgetary implications without input from central government. Larger decisions that would affect the budget would need to pass through the Ministry of Health | Approval comes from the regional health autority and parish office | approval for interventions made at the regional level | Approval for all activities done at the regional level | All activities must be approved by the region before implementati on | Inadequate support for community based interventions- approval for decisions made at district or regional level are not always forthcoming | Inadequate support for community based interventions- approval for decisions made at district or regional level are not always forthcoming |
